# Complete series method (CSM): a convenient method to reduce daily heterogeneity when evaluating the regeneration time (RT) of insecticide-treated nets (ITNs)

**DOI:** 10.1186/s13071-024-06323-4

**Published:** 2024-05-22

**Authors:** Aidi Galus Lugenge, Olukayode G. Odufuwa, Jilly Jackson Mseti, Johnson Kyeba Swai, Ole Skovmand, Sarah Jane Moore

**Affiliations:** 1 Vector Control Product Testing Unit, Environmental Health and Ecological Science Department, Ifakara Health Institute, P.O. Box 74, Bagamoyo, Tanzania; 2 School of Life Sciences and Bioengineering, The Nelson Mandela African Institution of Science and Technology (NM-AIST), P.O. Box 447, Arusha, Tanzania; 3 Vector Biology Unit, Epidemiology and Public Health Department, Swiss Tropical and Public Health Institute, Kreuzstrasse 2, Allschwil, 4123 Basel, Switzerland; 4 University of Basel, Petersplatz 1, 4001 Basel, Switzerland; 5 MRC International Statistics and Epidemiology Group, London School of Hygiene and Tropical Medicine (LSHTM), London, WC1E 7HT UK; 6 MCC47, Castelnau le Lez, France

**Keywords:** Insecticide-treated nets, ITN, Regeneration time, *Anopheles* mosquitoes, Cone bioassay, Resistant, Malaria

## Abstract

**Background:**

“Regeneration time” (RT) denotes the time required to obtain a stable mortality rate for mosquitoes exposed to insecticide-treated nets (ITNs) after three consecutive washes of a net in a day. The RT informs the wash interval used to artificially age ITNs to simulate their lifetime performance under user conditions (20 washes). RT was estimated following World Health Organization (WHO) longitudinal method (LM) procedures. Longitudinal evaluation may introduce heterogeneity due to mosquito batch variability, complicating RT determination. To overcome this, nets at each stage of regeneration (i.e., 1, 2, 3, 5 and 7 days post wash) were prepared in advance and refrigerated; then, a complete regeneration series was tested with a single mosquito batch on 1 testing day, completing four series over 4 days. This study compared the complete series method (CSM) against the LM.

**Methods:**

The overall heterogeneity in the methods for estimating RT of one incorporated alpha-cypermethrin and piperonyl butoxide (PBO) and one incorporated permethrin with PBO ITNs was determined using laboratory-reared resistant *Anopheles arabiensis* under standard laboratory conditions. LM methods and CSM were compared in two experiments with refrigerated nets acclimated for (i) 2 h (test 1) and (ii) 3 h (test 2). Four regeneration replicates per day were tested per ITN product with 50 mosquitoes exposed per replicate (equivalent sample size to LM). The heterogeneity from these methods was compared descriptively.

**Results:**

The intra-method variability for unwashed pieces was minimal, with variance of 1.26 for CSM and 1.18 for LM. For unwashed nets, LM had substantially greater variance and ratio of LM:CSM was 2.66 in test 1 and 2.49 in test 2. The magnitude of mortality measured in bioassays depended on sample acclimation after refrigeration.

**Conclusions:**

The CSM is a convenient method for determining the regeneration times. ITNs are prepared in advance, reducing pressure to prepare all samples to start on a single day. A complete regeneration series of samples is removed from the refrigerator, defrosted and evaluated on a single day with one mosquito batch reducing the influence of mosquito batch heterogeneity on results. Replicates can be conducted over several days but do not have to be conducted on consecutive days, allowing easy facility scheduling.

**Graphical Abstract:**

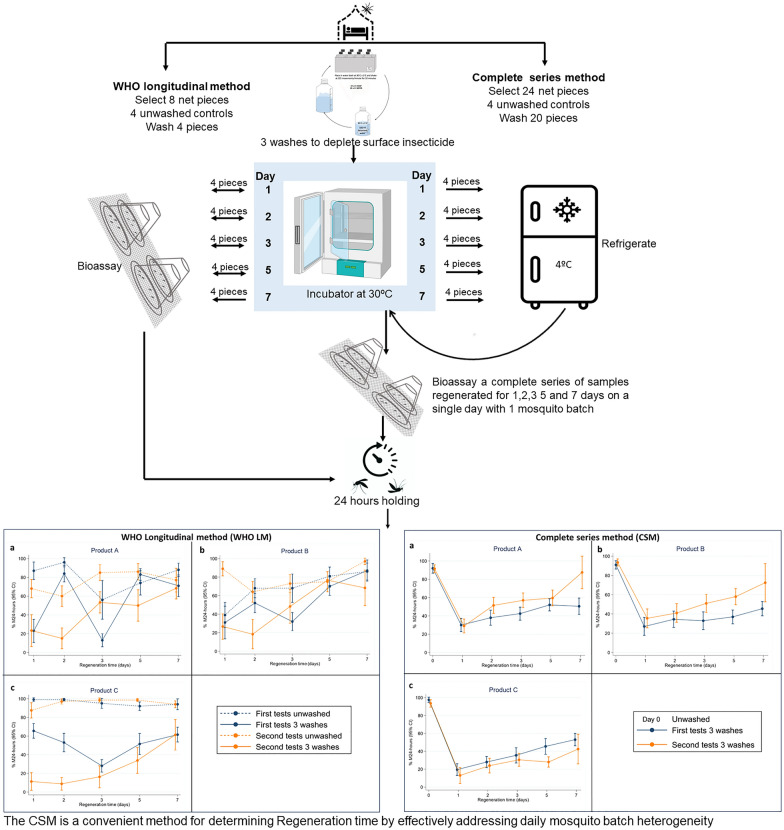

**Supplementary Information:**

The online version contains supplementary material available at 10.1186/s13071-024-06323-4.

## Background

Insecticide-treated nets (ITNs) are core tools for controlling malaria in endemic areas because they provide a physical barrier between potential human hosts and mosquitoes. The insecticides in ITNs, depending on their mode of action, induce mortality, knockdown, blood-feeding inhibition, irritancy and exiting and impact mosquito fertility [1]. When applied at scale, they reduce mosquito population size and average age [2], which lowers malaria transmission for the whole community [3, 4]. Between 2000 and 2015, ITNs averted approximately 68% of global malaria cases [5], and while the barrier effect reduces malaria among users, it is the insecticidal activity of ITNs that has the greatest impact on malaria [4]. The first generation of ITNs was impregnated with pyrethroid-based insecticides [6]. However, due to the increasing resistance of mosquitoes to pyrethroid insecticides [7, 8], dual-active ingredient (AI) ITNs including pyrethroid with additional AI including chlorfenapyr, pyriproxyfen and piperonyl butoxide (PBO) have been developed [9]. PBO inhibits metabolic enzymes that detoxify pyrethroids within mosquitoes, and ITNs treated with pyrethroids plus the synergist PBO are effective against mosquitoes that have evolved mechanisms to overcome pyrethroid insecticides through increased insecticide detoxification (metabolic resistance) [10].

For ITNs to be eligible for the donor-financed market, their efficacy must be demonstrated in a series of tests that measure the bioefficacy, chemical and physical quality of the nets in line with the guidelines of the World Health Organization Prequalification team (WHO-PQT) [11]. In the 2023 version of the guidelines, RT decisions were assessed based on chemical data although estimates from bioassays were still used to support the findings [11]. Bioefficacy testing is used to measure the biological efficacy of an AI against *Anopheles* mosquitoes and will be the primary focus of this study [12].

Among the methods used in evaluating ITNs, estimating regeneration time is critical because it is used to determine the wash interval of ITNs. Washing the ITN 20 times is used to artificially age the ITN, mimicking the loss of AI under user conditions. Artificially aged nets are then used in other studies, such as laboratory wash resistance and experimental hut studies, which are used to determine whether ITNs are sufficiently bio-efficacious throughout their lifespan to be PQ listed [11].

The RT measures the time required (in days) after washing for an ITN to regain its intended entomological effect. It characterises the time taken for the AI bound in the coating or yarn of an ITN to become biologically available on the surface of the yarn at a concentration sufficient to induce the expected effect on the mosquito used in the bioassay. This effect may be mortality, knock-down, reduced blood feeding or reduction in fertility [11]. This was estimated following the WHO longitudinal process: net samples were cut from the sides and roof of ITNs following a specific pattern: washed, rinsed and dried three times in a single day to remove the insecticide from the surface, after which the net samples were tested using cone bioassay at days 1, 2, 3, 5 and 7 post-wash following the 2013 WHO guidelines [12]. Several factors influence bioassay results [13] including ITN preparation exposure temperature [14] and test procedures [15]; mosquitoes also affect the results by their age [16], blood-feeding status [17], density [18], species [19], resistance level [20] and fitness [6, 21]. Mosquito heterogeneity and its impact on bioassays was first described in 1971 [22] and has been shown to impact insecticide susceptibility [21]. It is reduced but not eliminated by careful mosquito rearing [23].

Therefore, the present study aimed to minimise daily heterogeneity introduced by variability between mosquito batches. The study compared a new complete series method (CSM) against LM. The method involves washing, rinsing and drying of net pieces three times a day and allowing them to regenerate for a specific time in an incubator (0, 1, 2, 3, 5 or 7 days). On each day of regeneration, samples are removed from the incubator and stored in the refrigerator at 4 °C to stop further regeneration. Bioassays for a complete series of samples (0, 1, 2, 3, 5 and 7 days of regeneration) are then run together on a single day.

## Methods

### Study design

Single-blinded laboratory studies were conducted to estimate the regeneration times of two ITNs by WHO cone bioassay using (i) the standard longitudinal method (LM) following WHO guidelines [12] and (ii) the complete series method (CSM)—where a series of nets regenerated for 0, 1, 2, 3, 5 and 7 days post-wash were tested against the same batch of mosquitoes in a single day. ITNs were cut following WHO 2013 guidance [12] to maximize heterogeneity between samples. The LM and CSM bioassays were conducted twice to measure the best way to acclimate ITNs that have been refrigerated: (i) ITNs were acclimated for 2 h, 1 h in the incubator (30 °C) and 1 h at room temperature (27–29 °C) and experiments were conducted between 17:00 and 23:00 h (test 1). (ii) The ITNs were acclimated for 2 h in the incubator (30 °C) and 1 h at room temperature (27–29 °C) and experiments were conducted between 17:00 and 20:00 h (test 2).

### Testing facility

All tests were conducted at the Vector Control Product Testing Unit (VCPTU) [Good Laboratory Practice (GLP) accredited, South African National Accreditation System (SANAS) G0033] of the Ifakara Health Institute (IHI) located in Bagamoyo, Tanzania, from May to October 2023.

### Mosquito rearing

Laboratory-reared pyrethroid-resistant *Anopheles arabiensis* (Kingani strain) mosquitoes were used for cone bioassay tests. The mosquitoes were 2–5-day-old nulliparous females and sugar-fed. From May to October 2023, the mosquitoes were reared at temperatures between 26–29 °C and relative humidity between 59–83% with an ambient (approximately 12 h:12 h) light: dark cycle in line with MR4 Guidance [24]. Larvae were maintained at an average density of 200 per litre, fed ground Tetramin® fish flakes dispersed evenly across the top of the water using a spoon, and adult mosquitoes were provided 10% sterile (autoclaved) sucrose solution and were also provided with cow blood through membrane feeding to stimulate egg laying. *Anopheles arabiensis* (Kingani strain) was confirmed at the time of testing to phenotypically express resistance against all pyrethroid classes at 1 × discriminating concentration, that was fully restored with pre-exposure to PBO (Table S1).

### Test items

Two test items were used. One prototype 130-denier monofilament polyethylene incorporated ITN with a dosage of 12.95 g PBO/kg net and 2.72 g alphacypermethrin/kg net. The net is made from two yarns where one has a high dosage of PBO and a low of alphacypermethrin and the other yarn has the opposite dosage. The two yarns are exposed more or less on the two sides because of the knitting pattern and are here named products A and B: (i) Product A has mostly PBO yarn with 16.74 g/kg PBO and 0.62 g/kg alphacypermethrin. (ii) Product B has relatively more of the alphacypermethrin yarn with 1.27 g/kg PBO and 14.23 g/kg alphacypermethrin. (iii) Product C is a 150-denier monofilament polyethylene incorporated ITN with 20 g/kg permethrin and 10 g/kg PBO manufactured by Sumitomo Chemical, Japan [25]. A negative control net was used to assess the quality of the experiment: Safi Net, a polyester untreated net made of 75 denier monofilament fibres manufactured by A-to-Z Textile Mills, Tanzania.

### Study procedures

#### Net sampling

Four nets were selected per product. The nets were labelled using four-digit codes to ensure blinding of the testing team personnel and allow traceability of samples. Product A (front yarn) and B (back yarn) were identified with a small paper stapled at the edge of each sample.

##### Longitudinal method

**Fig. 1:**
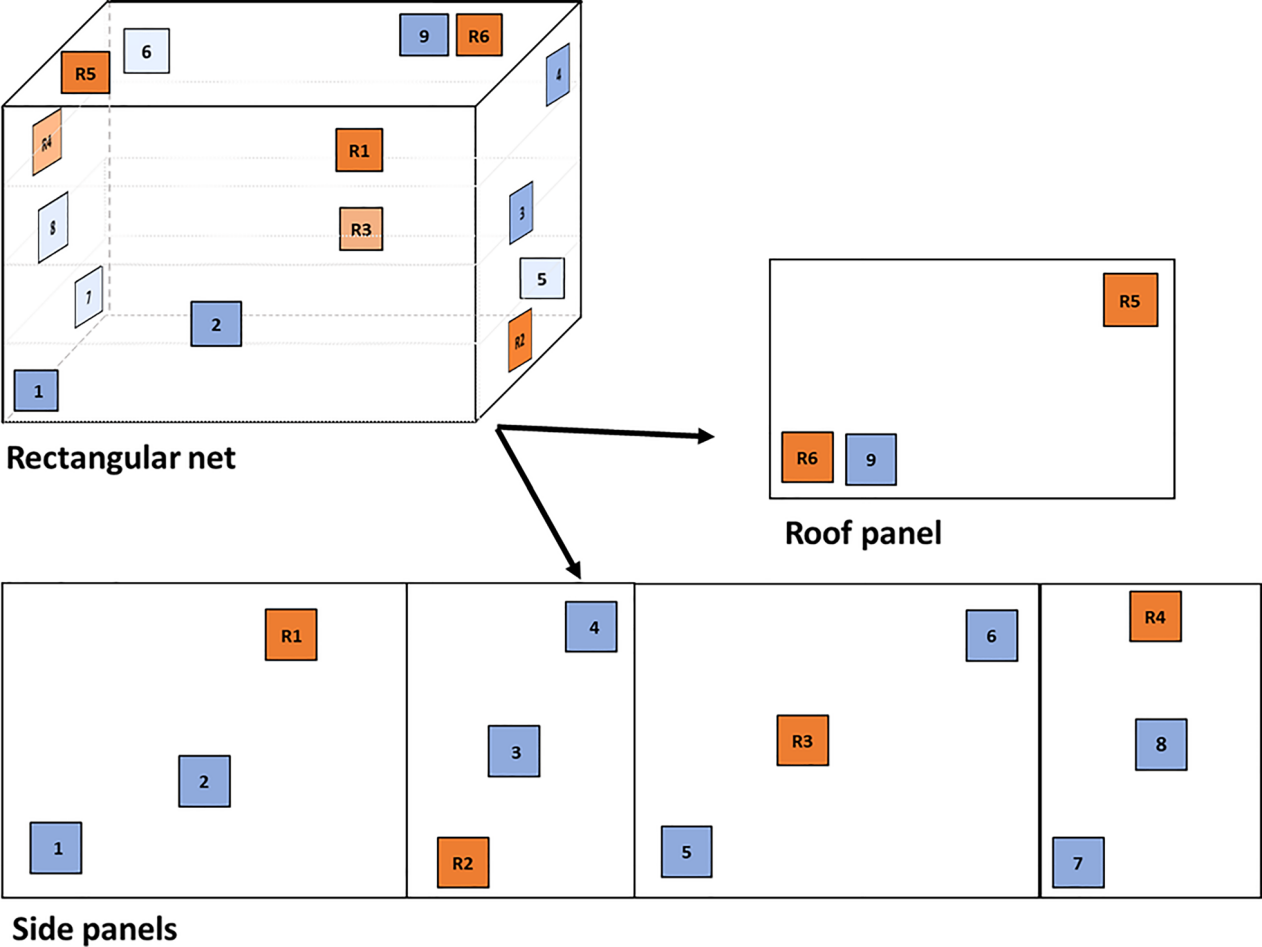
Insecticide-treated net (ITN) sampling scheme. Blue squares depict the nine positions from which samples are cut following WHO 2013 methodology. Two samples per net were selected at random for testing. Orange squares represent the six locations from which samples were cut for the complete series method. Samples were cut from all over the net to mimic the heterogeneity of sampling according to WHO 2013 methodology

Two net pieces (25 cm × 25 cm) were randomly selected from each of the four nets from positions 1–9 (a total of eight pieces per product) shown in blue in (Fig. 1). From each of the two pieces per net, one was washed, and one was left unwashed. After cutting, the pieces were wrapped in aluminium foil and stored in a refrigerator at 4 °C and acclimatised at 30 °C for 2 h before washing.

##### Complete series method

Six net pieces (25 cm × 25 cm) were cut from each of the four nets from positions R1–R6 (a total of 24 pieces per product) shown in orange in (Fig. 1). From each ITN, five pieces were washed, and one was unwashed. All the pieces were wrapped in aluminium foil, stored in a refrigerator at 4 °C and acclimatised at 30 °C for 2 h before washing.

#### Net washing

**Fig. 2:**
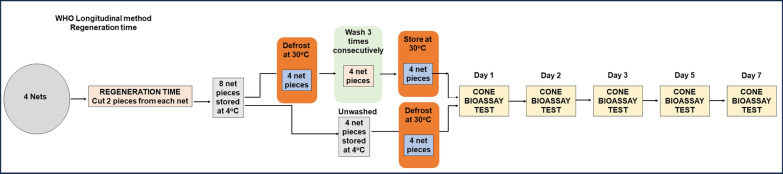
WHO Longitudinal method procedures for regeneration time washings and bioassays

**Fig. 3:**
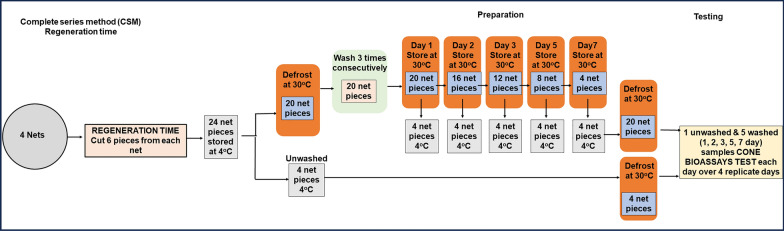
Complete series method procedures for regeneration time washings and bioassays

The net pieces cut for washing were individually introduced into 1-L glass bottles containing 500 mL palm soap (Jamaa® brand) solution (2 g/L) at 30 °C ± 2 °C. The bottles were capped and placed in an upright position in a Julabo SW22 water bath set at a 30 °C. Bottles were shaken for 10 min at 155 rpm, after which the pieces of netting were removed with tweezers, and the excess fluid was removed by gently shaking. After washing, the piece was rinsed twice in fresh deionized water. For each rinse, the net piece was added to a 1-L glass bottle containing 500 mL of fresh deionized water at 30 °C ± 2 °C and the same procedure was followed as the soap washing. After the second rinse, excess water was removed from the net sample, and the net pieces were then dried on a line for 30 min at room temperature (27–29 °C) out of direct sunlight. Once the samples were dry, the procedure was repeated two additional times (for a total of three washes per day). At the end of the third washing, the dry net samples were laid flat in aluminium foil and stored at 30 °C in an incubator (Memmert UFE400/G410.2367) until bioassay for LM [12] (Fig. 2) or selected for storage in the fridge for CSM (Fig. 3).

##### Longitudinal method

For the WHO longitudinal test, RT was determined using a total of eight net samples, of which four were unwashed and four were washed three times consecutively on a single day to deplete the insecticide on the net surface [12]. After drying, these net pieces were held at 30 °C in an incubator for testing on days 1, 2, 3, 5 and 7 post-washing (Fig. 2).

##### Complete series method

A total of 24 net samples were used, where 4 were unwashed and 20 net samples were washed three times consecutively on a single day to deplete the insecticide on the net surface and stored flat in the incubator at 30 °C after drying. At each of post-wash day 1, 2, 3, 5 and 7, four samples (1 sample per net) selected at random were removed from the incubator set at 30 °C and stored in the fridge set at 4 °C range (2–8 °C) until the last day when sample selection was completed. One set of samples selected to represent 1, 2, 3, 5 and 7 post wash were bioassayed together with the 0 day sample on 1 day consisting the complete series (Fig. 3). Four complete series were tested per net type over4 days so that all four samples were tested as used for LM.

#### Cone bioassays

Cone bioassays were performed according to standard WHO 2013 procedures [12], with two modifications to standardize exposure: (i) the boards were held at 60 degrees [26] and (ii) holes were cut in the board to ensure that the mosquitoes rested only on the net [15]. On each netting sample, cones were placed and held in place using masking tape. Five laboratory-bred mosquitoes (sugar fed, 2–5 day old) were introduced into each cone and exposed for 3 min. After exposure, the mosquitoes were removed gently from the cones and kept in paper cups (one cup per cone) provided with cotton wool moistened with 10% sugar solution. Mortality at 24 h (M24) after the end of the exposure period was recorded. Mosquitoes were determined to be alive if they were able to stand and fly and dead if mosquitoes were immobile or incapable of standing or flying after 24 h of exposure [12].

In the first test of the LM, bioassays were conducted over 7 consecutive days between 17:55 and 23:24 h with new mosquito batches on day 3 and day 7 (Table S3). The second tests of LM were conducted between 17:23 and 20:16 h with a new mosquito batch on day 3 (Table S3).

The CSM experiments were conducted over 4 consecutive days, with bioassays for the first and second test carried out from 17:20–20:25 h and 17:35–21:08 h (Table S3), respectively. In both test rounds a single batch of mosquitoes was utilized for a complete series of samples.

### Data analysis

The data were double entered into Excel and analysed with Stata 17.0 statistical software StataCorp [27]. Mortality in the negative control was low (< 10%); therefore, control-corrected mortality was not estimated [28]. Percentage arithmetic means with their 95% confidence intervals (CIs) for mortality were calculated. The total variance for each method was measured using the formula $S^{2} = \frac{{\sum {\left( {x - \overline{x}} \right)^{2} } }}{n - 1}$ summing variance for each point estimate (day) to determine total variance [29] and the ratios of the total variance between rounds using the same method (intra-method heterogeneity); the ratios of the total variance between methods within a single round (the inter-method heterogeneity) were alsocalculated. The difference in point estimates for each product and day of regeneration was estimated using a Wilcoxon signed rank test.

## Results

### Regeneration time curves

**Fig. 4:**
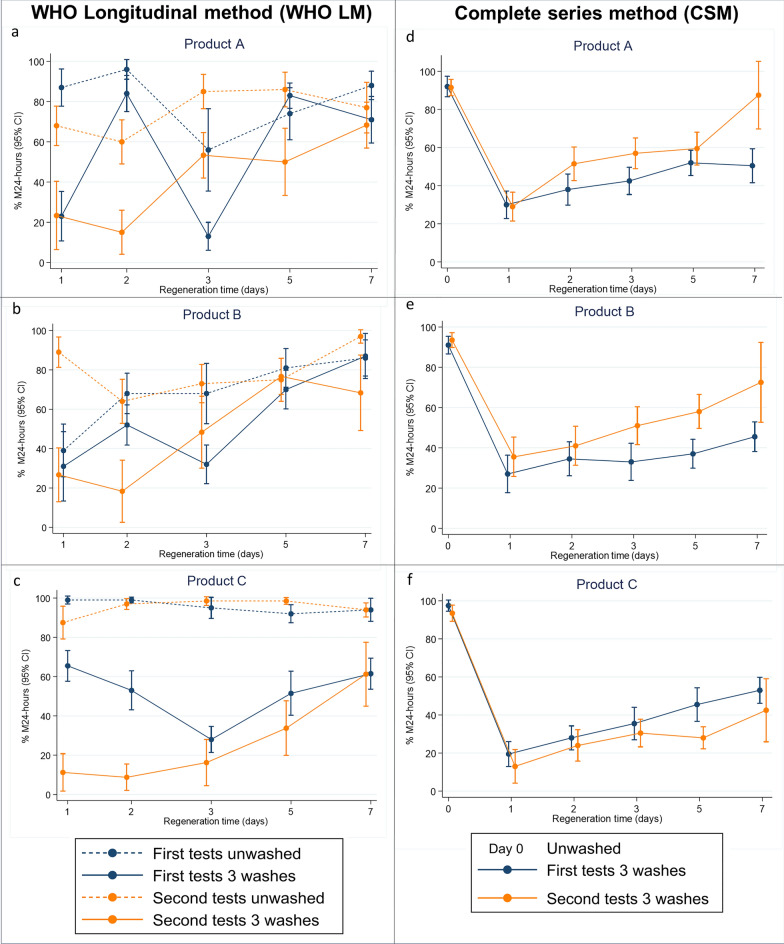
Regeneration time tests conducted with WHO cone tests against metabolically resistant *Anopheles arabiensis*. Tests measured control corrected 24-h mortality (M24) with 95% confidence interval (95% CI) of pyrethroid PBO ITNs using the standard WHO longitudinal method and complete series method, *N* = 40 cone tests per time point. Blue colour represent first test with 2 h acclimation and orange colour signifies the second test with 3 h acclimation for refrigerated samples. Solid lines represent washed ITNs, while dotted lines represent unwashed ITNs of the same product tested concurrently for reference. Product A had 16.74 g PBO and 0.62 g alphacypermethrin, Product B had 1.27 g PBO and 14.23 g alpha-cypermethrin and Product C with 150-denier monofilament polyethylene incorporated had 20 g permethrin and 10 g PBO

The regeneration process of all the products are presented in Fig. 4 where each dot represents a mean of ten cone test replicates for each tested net sample and four net samples tested at each time point (*N* = 40 cones). Mean 24-h mortalities for unwashed and washed net samples against resistant *An. arabiensis* (Kingani) for all methods, products and test rounds are presented in Table S2.

### Intra- and inter-method variances

The analysis showed the intra-method variance ratio for unwashed ITN pieces was 1.26 for LM and 1.18 for CSM, indicating similar levels of variability (around 20%) between replicate tests of either method (Table 1). The intra-method variance for washed nets indicated similar variability with a ratio of 1.04 for WHO LM and was less for CSM at 0.72 (28% difference between rounds).

**Table 1:** Intra-method heterogeneity

Intra-method variance measured as total variance from the sample mean for all samples				
Test 1 vs Test 2	Condition	Test 1	Test 2	Variance ratio
WHO longitudinal method	Unwashed	503	398	1.26
	Washed	1076	1031	1.04
Complete series method	Unwashed	189	160	1.18
	Washed	680	945	0.72

The total variance from the sample mean for all samples is calculated for each test and the difference between tests for a single method is expressed as a ratio

**Fig. 5:**
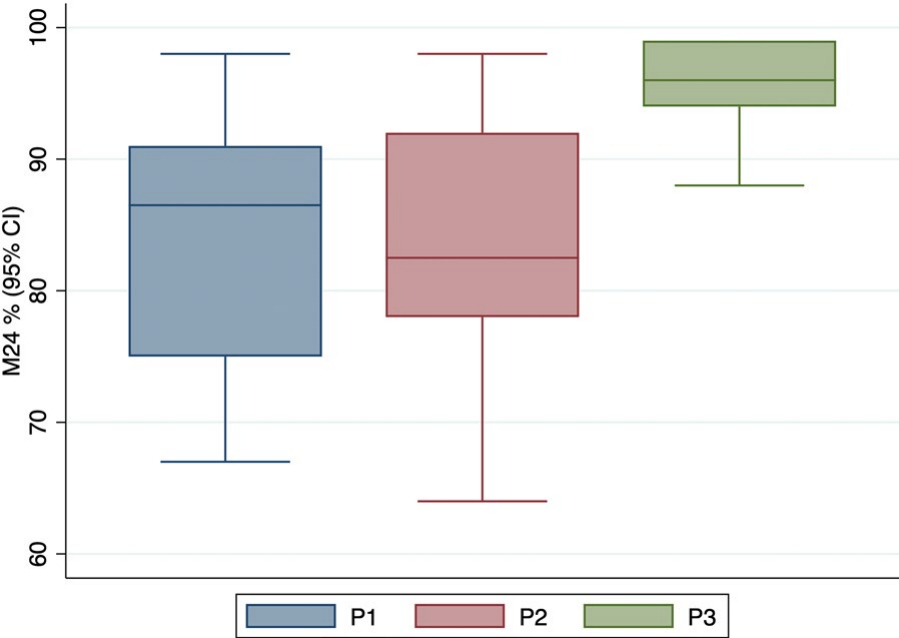
Range of point estimates of mosquito 24-h mortality measured in WHO cone tests against metabolically resistant *Anopheles arabiensis*. Tests measured control corrected 24-h mortality (M24) with 95% confidence interval (95% CI) of pyrethroid PBO ITNs as a positive control on each day that the WHO longitudinal method was conducted. Box plot represents median, interquartile range, high and low values measured, *N* = 40 cone tests per observation. Product A had 16.74 g PBO and 0.62 g alphacypermethrin, Product B had 1.27 g PBO and 14.23  alpha-cypermethrin and Product C had 150-denier monofilament polyethylene incorporated with 20 g permethrin and 10 g PBO

The daily heterogeneity in point estimates for unwashed ITNs is shown in Fig. 5. The point estimates varied by 30% between observations. Besides mosquito batch, possible sources of variation were temperature, relative humidity and time of test conduct (Table S3), although all efforts were made to control these variables and all were within allowable ranges as defined in WHO Guidance [30].

The WHO longitudinal method had higher variance for unwashed ITNs, which was almost double that of the complete series method in both tests (Table 2). The inter-method variability showed the variance ratio: 2.66 in the first test and 2.49 in the second test (Table 2).

**Table 2:** Inter-method heterogeneity

	Condition	Variance		
		WHO LM	Complete series	Variance ratio
Test 1	Unwashed	503	189	2.66
	Washed	1076	680	1.58
Test 2	Unwashed	398	160	2.49
	Washed	1031	945	1.09

The total variance of the sample mean for all samples is calculated for each test and the difference between methods for a single test run is expressed as a ratio

For washed nets variance was higher in the LM than CSM with a variance ratio of 1.58 in the first test and 1.09 in the second test. Much of this variance can be attributed to daily variance (Fig. 4).

**Fig. 6:**
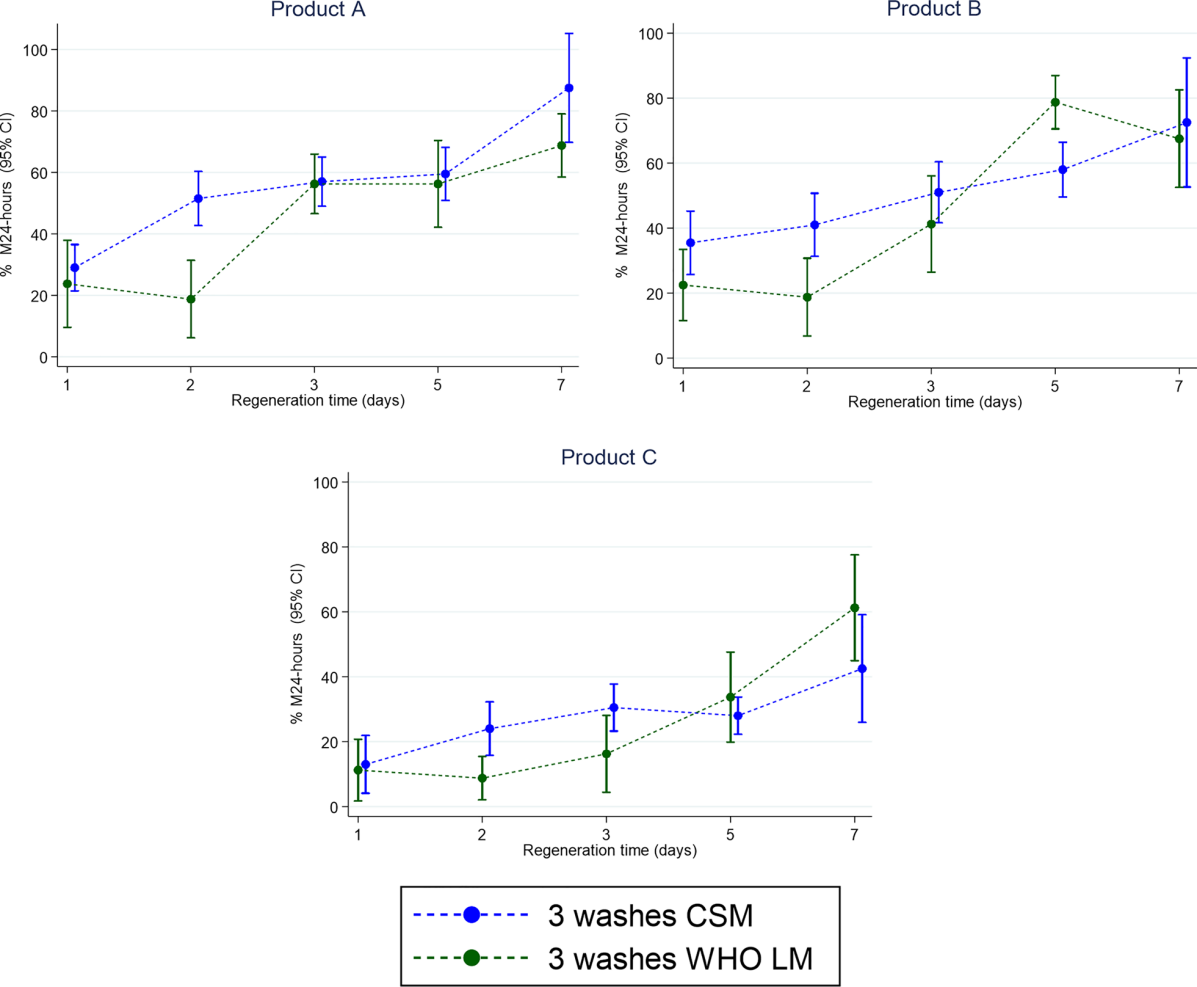
The second regeneration time tests conducted with WHO cone tests against metabolically resistant *Anopheles arabiensis*. Tests measured control corrected 24-h mortality (M24) with 95% confidence interval (95% CI): the graph indicates that complete series method (dotted blue line) and longitudinal method (dotted green line) in the second round yielded more comparable mortalities

The smaller difference in the variance ratio between methods in the second test was due to greater heterogeneity in CSM data when nets were acclimatized for 3 h while daily heterogeneity remained low. While variance was smaller in the first CSM test, the point estimates differed significantly from those in the LM (*z* = 2.244, *d.f.* = 29, *p* = 0.0221). Point estimates were not different between LM and CSM in the second round with longer ITN acclimation time (*z* = − 1.023, *d.f.* = 29, *p* = 0.3235). This can be seen in Fig. 6, where the regeneration curves for LM and CSM in round 2 are shown, highlighting the importance of correct ITN acclimation after refrigeration and before testing if using this method.

## Discussion

This study was conducted to determine how to reduce the heterogeneity commonly observed in regeneration time experiments caused by daily heterogeneity in mosquito batches. Fluctuations have been observed in multiple studies conducted at well-run laboratories [30–32] and were observed in this study very clearly on day 3 of the WHO LM when a new batch of mosquitoes was introduced, substantially skewing the results.

The design of the complete series method (CSM) study allowed a complete series to be run with one batch of mosquitoes, which substantially reduced the overall variation observed especially with repeat observations of unwashed ITNs. Even though the number of samples used in CSM (28) was higher than for the LM (4), and slight differences in AI concentration in different samples are another source of variation that may influence the precise estimate, the variation was still lower in CSM, indicating that other sources of variation are more important. This lead us to conclude that the reduced daily heterogeneity could be attributed to mosquitoes from the same batch (Table S4). It is notable that mortality rates fluctuated in repeated measurements of the same unwashed ITN pieces even though temperature, relative humidity and operators were carefully controlled. However, while the CSM reduced daily heterogeneity, inter-panel variation in dosage might have contributed to variations in the observed mortality because different pieces were tested each day [6]. This can be minimised by selecting pieces from the same band of the ITN. The variability in bioassays is an important concern and should be carefully considered when designing assays that rely on precise estimates [13].

It was observed that the intra-method variability of unwashed and washed ITN pieces was relatively low, approximately 20% for both methods between the first and second tests. The first assays were conducted over a longer time frame and later into the evening, which may have an impact on mosquito mortality since *Anopheles arabiensis* is nocturnal and its metabolism increases in the evening and can impact detoxification of the pyrethroid [33]. There are reports on circadian rhythm affecting the impact of metabolic detoxification of insecticides [33–35]. This may result in an overestimate of ITN mortality if pyrethroid PBO ITNs are tested outside of the dark phase [36]. Conversely, the effects of chlorfenapyr, a pro-insecticide, were more pronounced when tests were performed overnight when mosquitoes are metabolically active [37]. The second tests for both methods maintained consistent bioassay timing across all test nets.

The principle underlying the estimation of the RT is that following three consecutive washes in a single day to deplete the ITN surface of bioavailable AI, there is migration of AI from the core or binder to the surface of the ITN where it becomes bioavailable. This increase in bioavailable AI is reflected by an increase in the mortality measured in bioassays. Once the regeneration curve “flattens”, it indicates stability, suggesting that the mortality rate and consequently AI have reached a plateau or equilibrium [38]. The data therefore rely on precise estimates of mosquito mortality that can be obscured by daily heterogeneity introduced by testing parameters (test system, environmental conditions, operator bias). The data from this experiment indicate that daily heterogeneity is substantial despite tight control of testing conditions and mosquito rearing and is mainly driven by mosquito batch differences. Therefore, the CSM, which involves testing a single series of regenerating ITNs within a single day using a single batch of mosquitoes under equivalent conditions, contributed to the reduction of the variability observed.

Several factors could have affected mosquitoes between batches used, including differences in age [39] and fitness. Mosquito fitness is affected by variations in temperature [40] and density [21] during rearing. In this study, mosquitoes aged 2–5 days were used, and when the tests were conducted over a 7-day period in the WHO LM, mosquitoes from different batches were introduced, as a batch can only be used for 3 days to remain within this age range.

Temperature during testing [41, 42] and differences in skill between technicians (operator bias) [43] as well as mosquito density in cones [14] can affect measured mortality. A laboratory study revealed that the lack of reproducibility in results was attributed to the involvement of multiple inexperienced operators [44]. In this study, the WHO LM bioassays were conducted by several technicians possibly adding to variability but all other conditions were closely controlled. For the complete series method, the same technician was consistently involved in conducting one complete series of samples on a single day, enhancing the accuracy and reliability of the results.

All products during the second tests against the resistant strain demonstrated an increasing mortality until day 7 but had still not reached pre-wash levels, suggesting that RT did not stop by this day. Regeneration time studies were devised for pyrethroid ITNs using susceptible mosquito strains. However, the RT against resistant strains for the same product may be longer as the resistant strain requires a higher dose of insecticide to be killed [6]. Washing nets with too short intervals may result in little insecticide removal per wash, allowing a product to resist more washes. A previous study showed that when PermaNet was washed with 1-day interval, it gave > 80% mortality after 15 washes, but this declined to just 32% after 5 washes when wash interval was increased to 7 days [45]. According to the new WHO guidelines [11], longer regeneration can be employed in evaluating the efficacy of products and strain selection is carefully considered [46]. Therefore, it is hoped that more rigorous testing of products for prequalification will lead to enhanced long-term ITN performance.

The second round of the assays used a longer defrosting period, which increased the observed mortality measured. When evaluating ITNs containing liquid PBO or permethrin, it is likely to be affected by temperature and should be thoroughly defrosted (John Lucas, pers. comm). Therefore, careful and standardised preparation of net samples is required to ensure reproducible results for LM and CSM and any other study where ITN samples are refrigerated for preservation before testing as is commonly done in ITN-testing facilities. In this study, 2 h of defrosting samples laid flat between layers of foil in the incubator at 30 °C followed by an hour on the testing board was adequate to bring estimates of samples in the CSM in line with those of the WHO LM where samples were maintained at 30 °C between tests.

Apart from increasing the precision of estimates by reducing daily heterogeneity when making regeneration time curves, samples are prepared in advance over several days and refrigerated, allowing easier facility scheduling than if all samples have to be prepared to start on one day. If insufficient fit insects are available on a given day, there is no pressure to conduct the test using insects from a different source or of low fitness as there is with a scheduled longitudinal assay. Wash resistance tests were traditionally conducted following the WHO LM protocol, where a test sample was exposed to a repeated washing intervals defined by the RT test and then followed by bioassay after 0, 1, 2, 3, 5, 7, 10, 15 and 20 washes. This method is also exposed to day-to-day variations in the mosquito colony and can have the advantage of the CSM approach. According to this test, at least eight samples should be taken per wash test and to ensure that sampling methods are designed to minimize inter-sample variation. It is likely that a similar preparation of samples in advance and evaluation of a complete wash series will also lead to reduced variation and simpler scheduling.

The study identifies limitations arising from inconsistent timing of the experiment, suggesting the importance of conducting studies at a similar time each day with a short interval (within 2–3 h) to further reduce heterogeneity between observations. There is currently no study demonstrating a standardised acclimatisation time for conducting experiments and its potential impact on the condition of net samples, showing a need for further standardization as this clearly affected the measured mortality. Lastly, that the complete series method for regeneration time evaluations was conducted at a single facility potentially limits the generalisability of the results. Future studies are encouraged to investigate the robustness of the complete series method (CSM) across various facilities and products. It is a further limitation that sample size was not specifically investigated. It is likely that greater improvements to the RT curves could be attained by using greater replication.

## Conclusions

The complete series method (CSM) proved to be a convenient and robust method for reducing daily heterogeneity in bioassays as used in determining regeneration time resulting in more repeatable bioassay results. ITNs are prepared in advance following highly controlled standard washing procedures and allowed to regenerate in temperature-controlled incubators. Once samples regenerate to a specified day, they are refrigerated. If several products are to be tested on a single day, the preparation of multiple samples can be done in advance over a period of several weeks. Then, a single complete regeneration series of samples is removed from the refrigerator, defrosted and evaluated on a specified day using one mosquito batch per replicate. The total replicates can be conducted over a number of days but do not have to be conducted on consecutive days, allowing easy facility scheduling.

### Supplementary Information

Supplementary Material 1. **Table S1.** Resistance profile of *Anopheles arabiensis* mosquitoes measured by mortality at 24 h (M24) (WHO susceptibility last test conducted in Q1, January 2023).

Supplementary Material 2. **Table S2. **Observed regeneration time using WHO procedure: 24-h mortality for *Anopheles arabiensis* (Kingani strain, resistant) exposed to ITN samples: unwashed longitudinal (UW-L), washed three times longitudinal (WL) and unwashed (UW-C), washed complete series (WC) at 1, 2, 3, 5 and 7 days after washing in cone bioassay.

Supplementary Material 3. **Table S3.** Details of the testing conditions for the bioassays.

Supplementary Material 4. **Table S4**. The total variances from the sample mean for all unwashed samples were calculated for each test day. Unwashed samples were used to calculate daily heterogeneity from the mosquito batches and not differences in surface chemistry during regeneration.

Supplementary Material 5. Dataset S1. WHO longitudinal method first round.

Supplementary Material 6. Dataset S2. WHO longitudinal method second round.

Supplementary Material 7. Dataset S3. Complete series method first round.

Supplementary Material 8. Dataset S4. Complete series method second round.

## Data Availability

The data generated in this study are available as follows: Additional file 5: contains dataset 1 for WHO Longitudinal method in the first round, Additional file 6: contains dataset 2 for WHO Longitudinal method in the second round, Additional file 7: Contains dataset 3 for Complete series method in the first round and Additional file 8: contains dataset 4 for Complete series method in the second round.
