# Supplementary material for: Complete series method (CSM): a convenient method to reduce daily heterogeneity when evaluating the regeneration time (RT) of insecticide-treated nets (ITNs)
Source: Parasit Vectors. 2024 May 22;17:235. doi: 10.1186/s13071-024-06323-4 (PMC11110420; doi:10.1186/s13071-024-06323-4)
Supplement: Supplementary file 1 — Supplementary Material 1. Table S1. Resistance profile of Anopheles arabiensis mosquitoes measured by mortality at 24 h (M24) (WHO susceptibility last test conducted in Q1, January 2023). [file 13071_2024_6323_MOESM1_ESM.docx]

| Strain | Permethrin (0.75%) | Deltamethrin  (0.05%) | 𝛼-cypermethrin (0.05%) | 𝜆-cyhalothorin | Pirimiphos methyl  (0.25%) | Bendiocarb  (0.1%) | PBO  (4%) | Permethrin (0.75%) + PBO (4%) | Deltamethrn  (0.05%) + PBO (4%) | 𝛼-cypermethrin (0.05%) + PBO  (4%) | 𝜆–cyhalothorin + PBO (4%) |
| --- | --- | --- | --- | --- | --- | --- | --- | --- | --- | --- | --- |
| *An. arabiensis*  (Kingani) | 18% | 29% | 18% | 7% | 100% | 100% | 3% | 100% | 100% | 100% | 100% |
