# Supplementary material for: Complete series method (CSM): a convenient method to reduce daily heterogeneity when evaluating the regeneration time (RT) of insecticide-treated nets (ITNs)
Source: Parasit Vectors. 2024 May 22;17:235. doi: 10.1186/s13071-024-06323-4 (PMC11110420; doi:10.1186/s13071-024-06323-4)
Supplement: Supplementary file 2 — Supplementary Material 2. Table S2. Observed regeneration time using WHO procedure: 24-h mortality for Anopheles arabiensis (Kingani strain, resistant) exposed to ITN samples: unwashed longitudinal (UW-L), washed three times longitudinal (WL) and unwashed (UW-C), washed complete series (WC) at 1, 2, 3, 5 and 7 days after washing in cone bioassay. [file 13071_2024_6323_MOESM2_ESM.docx]

| Round | Test product | Days after wash | 0 | 1 | | | 2 | | | 3 | | | 5 | | | 7 | | |
| --- | --- | --- | --- | --- | --- | --- | --- | --- | --- | --- | --- | --- | --- | --- | --- | --- | --- | --- |
|  |  | Condition | UW-C | UW-L | W-L | W-C | UW-L | W-L | W-C | UW-L | W-L | W-C | UW-L | W-L | W-C | UW-L | W-L | W-C |
| 1 | A | Exposed (n) | 200 | 200 | 200 | 200 | 200 | 200 | 200 | 200 | 200 | 200 | 200 | 200 | 200 | 200 | 200 | 200 |
|  |  | No dead | 195 | 182 | 47 | 39 | 196 | 164 | 56 | 150 | 25 | 71 | 153 | 153 | 91 | 184 | 146 | 106 |
|  |  | Mortality  (95% CI) | 92  (87,97) | 91  (86, 96) | 24  (16, 31) | 30  (23,37) | 98  (96,100) | 82  (76, 88) | 38  (30,46) | 75  (63, 87) | 13  (7, 18) | 43  (35,50) | 77  (70, 84) | 77  (71, 83) | 52  (45,59) | 92  (88, 96) | 73  (66, 80) | 51  (42,59) |
|  | B | Exposed (n) | 200 | 200 | 200 | 200 | 200 | 200 | 200 | 200 | 200 | 200 | 200 | 200 | 200 | 200 | 200 | 200 |
|  |  | No dead | 182 | 128 | 62 | 54 | 165 | 94 | 69 | 155 | 61 | 66 | 172 | 138 | 74 | 185 | 176 | 91 |
|  |  | Mortality  (95% CI) | 91  (87,95) | 64  (53, 75) | 31  (21, 41) | 27  (18,36) | 83  (76, 89) | 47  (40, 54) | 35  (26,43) | 78  (69, 86) | 31  (24, 38) | 33  (24,42) | 86  (80, 92) | 69  (63, 75) | 37  (30,44) | 93  (88, 97) | 88  (82, 94) | 46  (38,53) |
|  | C | Exposed (n) | 200 | 200 | 200 | 200 | 200 | 200 | 200 | 200 | 200 | 200 | 200 | 200 | 200 | 200 | 200 | 200 |
|  |  | No dead | 195 | 198 | 131 | 39 | 198 | 106 | 56 | 190 | 56 | 71 | 184 | 103 | 91 | 188 | 123 | 106 |
|  |  | Mortality  (95% CI) | 98  (95,100) | 99  (97, 100) | 66  (58, 73) | 20  (13,26) | 99  (98, 100) | 53  (43, 63) | 28  (22,34) | 95  (90, 100) | 28  (22, 34) | 36  (27,44) | 92  (88, 96) | 52  (41, 62) | 46  (37,54) | 94  (88, 100) | 62  (54, 69) | 53  (46,60) |
| 2 | A | Exposed (n) | 200 | 200 | 200 | 200 | 200 | 200 | 200 | 200 | 200 | 200 | 200 | 200 | 200 | 200 | 200 | 200 |
|  |  | No dead | 183 | 150 | 47 | 58 | 133 | 33 | 103 | 178 | 107 | 114 | 179 | 131 | 119 | 167 | 139 | 143 |
|  |  | Mortality  (95% CI) | 92  (87,96) | 75  (68,82) | 24  (16,31) | 29  (22,37) | 67  (59,74) | 17  (11,22) | 52  (43,60) | 89  (84,94) | 54  (48,59) | 57  (49,65) | 90  (84,95) | 66  (57,74) | 60  (51,68) | 84  (76,91) | 70  (64,75) | 88  (70,100) |
|  | B | Exposed (n) | 200 | 200 | 200 | 200 | 200 | 200 | 200 | 200 | 200 | 200 | 200 | 200 | 200 | 200 | 200 | 200 |
|  |  | No dead | 187 | 183 | 61 | 71 | 144 | 41 | 82 | 162 | 93 | 102 | 163 | 155 | 116 | 196 | 128 | 139 |
|  |  | Mortality  (95% CI) | 94 (90,97) | 92  (87,96) | 31 (21,40) | 36 (26,45) | 72  (65,80) | 21  (14,27) | 41 (31,51) | 81  (75,87) | 47  (37,56) | 51 (42,60) | 82 (75,88) | 78 (73,83) | 58  (50,66) | 98 (96,100) | 64  (56,72) | 73  (53,92) |
|  | C | Exposed (n) | 200 | 200 | 200 | 200 | 200 | 200 | 200 | 200 | 200 | 200 | 200 | 200 | 200 | 200 | 200 | 200 |
|  |  | No dead | 187 | 175 | 29 | 26 | 194 | 18 | 48 | 197 | 29 | 61 | 197 | 89 | 56 | 188 | 122 | 104 |
|  |  | Mortality  (95% CI) | 94 (89,98) | 88  (79,96) | 15  (9,20) | 13  (4,20) | 97 (94,100) | 9  (5,13) | 24 (16,32) | 99 (96,100) | 15  (8,21) | 31 (23,38) | 99 (97,100) | 45 (36,53) | 28  (22,34) | 94  (91,98) | 61  (53,70) | 43  (26,59) |

Note: data presented as ***Arithmetic Mean (95% Confidence interval), Control mortality was <10%***
