# Supplementary material for: Complete series method (CSM): a convenient method to reduce daily heterogeneity when evaluating the regeneration time (RT) of insecticide-treated nets (ITNs)
Source: Parasit Vectors. 2024 May 22;17:235. doi: 10.1186/s13071-024-06323-4 (PMC11110420; doi:10.1186/s13071-024-06323-4)
Supplement: Supplementary file 3 — Supplementary Material 3. Table S3. Details of the testing conditions for the bioassays. [file 13071_2024_6323_MOESM3_ESM.docx]

| Method | WHO Longitudinal method (WHOLM) | | | | | | | | | | Complete series method (CSM) | | | | | | | |
| --- | --- | --- | --- | --- | --- | --- | --- | --- | --- | --- | --- | --- | --- | --- | --- | --- | --- | --- |
| Test round | 1 | | | | | 2 | | | | | 1 | | | | 2 | | | |
| Day | 1 | 2 | 3 | 5 | 7 | 1 | 2 | 3 | 5 | 7 | 1 | 2 | 3 | 4 | 1 | 2 | 3 | 4 |
| Date of replicate | 29May | 30May | 31May | 02Jun | 04Jun | 02 Oct | 03 Oct | 04 Oct | 06 Oct | 08 Oct | 09Jun | 10Jun | 11Jun | 14Jun | 25Sep | 26Sep | 27Sep | 29Sep |
| Temperature  Median (IQR) | 27  (27-28) | 27  (26-27) | 27  (26-27) | 26  (26-27) | 27  (27-27) | 29  (29-30) | 29  (29-29) | 29  (29-29) | 29  (29-30) | 29  (29-30) | 27  (27-27) | 27  (27-27) | 27  (27-27) | 27  (27-27) | 28  (28-28) | 28  (27-28) | 28  (28-28) | 28  (28-29) |
| Humidity  Median (IQR) | 82  (81-83) | 79  (76-84) | 67  (65-70) | 76  (75-86) | 76  (75-85) | 74  (68-79) | 78  (79-80) | 80  (78-80) | 78  (74-83) | 78  (74-79) | 76  (76-77) | 75  (73-78) | 71  (66-75) | 71  (70-72) | 76  (75-77) | 79  (78-80) | 79  (79-80) | 78  (76-80) |
| Start time  (min-max) | 19:08-23:21 | 18:28-20:42 | 17:55-21:19 | 18:05-20:56 | 18:19-20:46 | 17:46-19:54 | 17:39-19:42 | 17:23-19:27 | 17:46-19:40 | 18:07-20:13 | 17:31-20:07 | 17:20-20:22 | 17:51-19:24 | 18:05-19:59 | 18:51-21:05 | 18:38-20:36 | 18:14-20:14 | 17:35-19:06 |
| End time  (min-max) | 19:11-23:24 | 18:31-20:45 | 17:58-21:22 | 18:08-20:59 | 18:22-20:49 | 17:49-19:57 | 17:42-19:45 | 17:26-19:30 | 17:49-19:43 | 18:10-20:16 | 17:57-20:10 | 17:43-20:25 | 18:15-19:27 | 18:35-20:02 | 19:33-21:08 | 19:12-20:39 | 18:52-20:17 | 17:38-18:51 |
| Technician | ISK&  ISM | ISK, ISM & DEK | ISK&  MPR | ISK &ISM | ISK&  MRP | ISM & ISK | ISM | ISM & MRP | ISM&  ISK | ISM & | MSA & MRP | MRP | ISM&  ISK | ISM &MRP | ISM | ISM & ISK | ISM & ISK | ISM& MSA |

*All experiments were conducted in 2023*
