# Supplementary material for: Complete series method (CSM): a convenient method to reduce daily heterogeneity when evaluating the regeneration time (RT) of insecticide-treated nets (ITNs)
Source: Parasit Vectors. 2024 May 22;17:235. doi: 10.1186/s13071-024-06323-4 (PMC11110420; doi:10.1186/s13071-024-06323-4)
Supplement: Supplementary file 4 — Supplementary Material 4. Table S4. The total variances from the sample mean for all unwashed samples were calculated for each test day. Unwashed samples were used to calculate daily heterogeneity from the mosquito batches and not differences in surface chemistry during regeneration. [file 13071_2024_6323_MOESM4_ESM.docx]

| Total variance from the unwashed samples | | | | | | | | | |
| --- | --- | --- | --- | --- | --- | --- | --- | --- | --- |
| Method | WHO Longitudinal method | | | | | Complete series method | | | |
| Day | **1** | **2** | **3** | **5** | **7** | **1** | **2** | **3** | **4** |
| Test 1 | 141 | 47 | 170 | 76 | 48 | 29 | 96 | 21 | 33 |
| Test 2 | 97 | 111 | 56 | 59 | 51 | 30 | 30 | 53 | 28 |
